# Differential mitochondrial DNA copy number in three mood states of bipolar disorder

**DOI:** 10.1186/s12888-018-1717-8

**Published:** 2018-05-25

**Authors:** Dong Wang, Zongchang Li, Weiqing Liu, Jun Zhou, Xiaoqian Ma, Jinsong Tang, Xiaogang Chen

**Affiliations:** 1 Department of Psychiatry, the Second Xiangya Hospital, Central South University, Changsha, Hunan China; 2 Laboratory of Medical Genetics, School of Life Sciences, Central South University, Changsha, Hunan China; 3 Department of Psychiatry, First Affiliated Hospital of Kunming Medical University, Kunming, Yunnan China; 4 Mental Health Institute, the Second Xiangya Hospital, Central South University, Changsha, Hunan China; 5 National Clinical Research Center on Mental Disorders, Changsha, Hunan China; 6 National Technology Institute on Mental Disorders, Changsha, Hunan China; 7 Hunan Key Laboratory of Psychiatry and Mental Health, Changsha, Hunan China

**Keywords:** Bipolar disorder, Mitochondrial DNA copy number, Mania, Depression

## Abstract

**Background:**

Accumulating evidences indicated that mitochondrial abnormalities were associated with bipolar disorder. As a sensitive index of mitochondrial function and biogenesis, Mitochondrial DNA copy number (mtDNAcn) may be involved in the pathophysiology of bipolar disorder.

**Methods:**

Leukocyte relative mtDNAcn was measured by quantitative polymerase chain reaction in subjects with BD (*n* = 131) in manic, depressive, and euthymic symptoms. Thirty-four healthy individuals were used as comparison control. BD clinical symptomatology was evaluated by Young Mania Rating Scale (YMRS), Hamilton Depression Scale (HAM-D), Clinical Global Impression-Bipolar Disorder-Severity of Illness Scale (CGI-BD-S), and the Positive and Negative Syndrome Scale (PANSS).

**Results:**

Compared to healthy controls, BD patients with manic and depressive symptoms presented significantly decreased mtDNAcn levels (*p*-value = 0.009 and 0.041, respectively). No significant differences were detected in mtDNAcn between euthymic patients and healthy controls. The mtDNAcn was negatively correlated with the number of relapses in manic patients (β = − 0.341, *p* = 0.044).

**Conclusions:**

Our study described the first evidence of (1) a significant decline of mtDNAcn in manic BD patients, (2) a similar decreased level of mtDNAcn between manic and depressed BD patients, (3) a negative correlation of mtDNAcn with number of relapses in patients suffering from manic states. Alterations of mtDNAcn in manic and depressed patients, which may reflect disturbances of energy metabolism, supported the role of mitochondrial abnormalities in the pathophysiology of BD.

**Electronic supplementary material:**

The online version of this article (10.1186/s12888-018-1717-8) contains supplementary material, which is available to authorized users.

## Background

Bipolar disorder (BD) is a chronic and several psychiatric illness, characterized by recurrent episodes of mania and depression, and interspersed with remission period. Although many systems and pathways have been involved in the pathophysiology of BD together containing signal transduction pathways, various neurotransmitter abnormalities, mitochondrial and metabolic dysfunctions and so on [1]; the definitive mechanisms of BD remains to be fully elucidated.

Mitochondria serve as key organelles in eukaryotic cells, well known for synthesizing adenosine triphosphate (ATP) from glucose by oxidative phosphorylation for energy production. Especially, the energy source of neurons relies primarily on mitochondrial oxidative phosphorylation. Additionally, mitochondria also play a vital role in calcium signaling, cell resilience, apoptosis, the regulation of reactive oxygen species (ROS) and DNA damage [2–4]. Mitochondrial DNA (mtDNA) is a double-stranded DNA molecule with no introns and histones. Without protection of histones and due to limited DNA repair capacity, these mtDNA is very prone to oxidative or genotoxic injury. As a result, mitochondria may increase their own copy number to compensate for the defects or impairment. Excessive oxidative stresses in pathological conditions may induce structural and functional changes of mitochondrial [5]. Postmortem, brain imaging, genetic and peripheral cells studies all support that mitochondrial dysfunction is associated with BD [6–10]. Using magnetic resonance spectroscopy, some studies showed lower level of phosphocreatine, N-acetyl-aspartate and inorganic phosphate and inorganic phosphate, which are supposed to reflect impaired mitochondrial function in several brain regions of living BD patients [6, 7]. There were lines of evidences revealing abnormal mitochondrial morphology in neurons of postmortem brain and peripheral cells of BD patients [8, 9]. Additionally, many SNPs across the genome of mitochondria were associated with BD [10, 11]. These observed changes maybe indicate an important role of mitochondria in the pathogenesis of BD.

Mitochondrial DNA copy number (mtDNAcn) that reflects the mitochondrial function and biogenesis could be measured per cell [12]. Abnormal mtDNAcn has been suggested to associate with various psychiatric disorders [13–15]. With regard to BD, few studies were conducted to address the possible role of mtDNAcn in BD. Vawter et al. firstly found the alteration of mtDNAcn in a post-mortem study [16], but other three post-mortem studies got negative results [17–19]. In peripheral blood leukocytes, Chang et al. revealed that euthymic BD patients had a lower mtDNAcn than control group [20]; While de Sousa et al. found no significant differences in mtDNA content between depressed BD patients and health controls [21]. Furthermore, the mtDNA content still did not have a significant decrease after lithium carbonate treatment.

Mitochondrial dysfunction may be a cause of BD symptoms. Given the heterogeneity of the disease itself and the inconsistent results from studies, it is necessary to find more evidence to interpret the effect of mitochondrial dysfunction in BD. Human leukocytes have been proved to reflect virtually the plasma oxidative damage to DNA [22]. Heretofore, no studies have assessed the mtDNA content in leukocytes of patients suffering mania or different states of BD. In this study, mtDNAcn was measured in BD patients with three different states and healthy individuals to identify how mtDNA content is altered in different mood states. We hypothesized that mtDNAcn variation may associate with BD, and would differentiate mood states as well as bipolar euthymia from healthy controls.

## Methods

### Participants

The study was approved by the ethics committee of the Second Xiangya Hospital of Central South University. All subjects voluntarily participated in the research and signed the informed consent. These procedures were performed in accordance with Declaration of the Helsinki. Acute BD patients were recruited from the inpatient and outpatient departments of the Second Xiangya Hospital in Hunan Province of China. The Euthymic patients of BD were primarily recruited from the outpatient clinic.

BD type I is defined by a pattern of depressive episodes and manic episodes. Subjects with BD type I were diagnosed by two or more trained psychiatrists using the Structured Clinical Interview for DSM- IV Axis I Disorders (SCID-IV) and its researcher version with psychotic screen (SCID-I/P). The exclusion criteria for BD patients included electroconvulsive therapy within past 3 months; substance abuse; other mental illness; neurological diseases; serious medical conditions; positive pregnancy test or lactation. Meanwhile, healthy volunteers were recruited by advertisements from local communities. Healthy controls with substance use, history of mental or neurological disorders, serious medical disorders, positive pregnancy test or lactation were excluded from the study (see Additional file 1).

### Assessment

One hundred thirty-one subjects with BD type I and 34 healthy volunteers were enrolled in the study. There were 55 patients meeting the Diagnostic and Statistical Manual of Mental Disorders Fourth Edition (DSM-IV) criteria suffering from manic episodes, 47 patients in depressive episodes and 29 patients in euthymic states. Young Mania Rating Scale (YMRS), Hamilton Depression Scale (HAM-D), Clinical Global Impression-Bipolar Disorder-Severity of Illness Scale (CGI-BD-S) were used to assess the symptom severity respectively. Euthymic BD patients were clinically stable for more than 6 months and the index score for CGI-BD-S ≤ 2. When all patients were recruited in the study, the psychiatric assessment and blood collection would be completed in next 24 h.

### Assays

Blood samples (3 ml) were collected in EDTA tubes from the 165 participants and kept at − 80 °C. Genomic DNA was extracted from 200 μl blood samples following the manufacturer’s protocol using QIAamp DNA Mini Kit (Qiagen, Hilden, Germany). The quantity and purity of the DNA were measured by Nanodrop 2000 spectrophotometer.

The detailed methods and procedures were described in our previous research [13]. In brief, the mtDNAcn was evaluated by the ratio of the amount of mtDNAcn (ND1) to a reference nuclear single copy gene (hemoglobin subunit beta HBB) copy number. The primers for ND1 were as follow: F (5-CCCTAAAACCCGCCACATCT-3) and R (5-GAGCGATGGTGAGAGCTAAGGT-3). The primers for (HBB) were F (5-GTGCACCTGACTCCTGAGGAGA-3) and R (5-CCTTGATACCA ACCTGCCCAG-3).

The assays were conducted by using LightCycler® 480 SYBR Green I Master on a Roche LightCycler® 480 machine (Roche, Manheim, Germany). The double-standard curves method of relative quantification PCR was employed, which is an improved, simple, accurate method [23]. The quantitative assay was based on amplification using conditions, primer sequences according to Xing et al. [24]. PCR cycling condition of ND1 gene was 95 °C 10 min for 1 cycle, 30 cycles of 95 °C for 15 s, 60 °C for 1 m. The cycling condition of HBB gene was 95 °C 10 min, 40 cycles of 95 °C 15 s, 56 °C for 1 m. Each sample was run in triplicate using 10 ng DNA templates in a 10 ul reaction volume and randomly assigned to 96-well plates. The ND1 and HBB genes PCR reactions were performed on separate plates with the same samples located at the same positions., The measures of mtDNAcn for each sample were obtained by the ratio of ND1 copy number to HBB copy number from standard curves. The ratio was normalized to a calibrator DNA in order to standardize between different independent assays. The calibrator DNA sample was from a health control individual and used for comparison with results of different runs. The calibrator DNA sample was diluted 1:4 to build a five-point standard curve between 0.0195 and 20 ng/ul. The amplification efficiencies of the standards were between 92.3 and 108.2%. A negative control and a calibrator DNA were enrolled in each run. The inter-assay coefficient of variation (CV) was 0.15–3.5%, and the intra-assay CV was 1.13–4.37%.

### Statistical analysis

Statistical analysis was conducted using Minitab 16 (Minitab Inc., Pennsylvania) and SPSS 23.0 (SPSS Inc., Chicago, IL) for windows. Dichotomous categorical variables in groups were compared using Chi-square test. Continuous variables with normal distribution were analyzed using T test or one-way analysis of variance analysis (ANOVA) test. The mtDNAcn in four groups were skewed distributed, and then adapted into normal distribution using Box-Cox transformation in Minitab software [25]. The Box-Cox (1964) transformation model in Minitab could select the best mathematical function to obtain a normal distribution of the transformed data [26]. The primary outcome analysis was conducted using ANOVA test. When significance was shown in ANOVA, Generalized linear model and Bonferroni post hoc test were performed to examine the intergroup differences of mtDNAcn, after adjusting sexuality, age, years of education, body mass index (BMI). Effect sizes were calculated using Cohen’s eta squared η^2^ in ANOVA test and R^2^ in multiple regression analyses [27]. Cohen J suggested effect sizesη^2^ of < 0.01 were small, 0.06 medium, and ≥ 0.14 large. For each BD group, multiple regression analyses were used to explore the association between mtDNAcn and other variables. Descriptive statistics were shown as the mean ± standard deviation or median (interquartile range), and significance level was set at *P* < 0.05 (two-tailed).

## Results

### Demographic and clinical data of the sample

Table 1 lists demographic, clinical and medication information, and the mtDNAcn median (range) of the study sample. BD patients and healthy controls did not differ in terms of age, gender, BMI, months of education (all *P* > 0.05). Second generation antipsychotic drugs were prescribed for BD patients in the research, and their doses were converted to CPZ equivalents available [28]. As showed, there were no significant differences in using CPZ equivalents, anticonvulsants, benzodiazepines and Lithium (all *P* > 0.05). Manic subjects demonstrated more severe symptoms than depressive and euthymic subjects on Young Mania Rating Scale; and depressed group showed significantly higher HDRS scores than manic and euthymic groups. To identify effect of illness history on mtDNAcn, the detailed information about duration of illness and number of previous episodes were recorded. Both Length of illness and number of previous episodes were similar in three groups (*P* > 0.05).

**Table 1:** Demographic, clinical and medication information of bipolar patients and healthy controls

	Bipolar patients			Health control	Statistic		
	Manic *N* = 55	Depression *N* = 47	Euthymic *N* = 29	*N* = 34			
Age, mean (SD)	26.67 (7.15)	26.94 (8.91)	23.76 (3.94)	24.50 (3.82)	ANOVA, F = 2.05, *P* = 0.108		
Sex							
Male	26	23	9	19			
Female	23	24	20	15	df = 3, X^2^ = 4.13, *P* = 0.25		
Years of education, mean (SD)	12.55 (2.80)	12.38 (3.47)	12.24 (2.52)	13.56 (2.78)	ANOVA, F = 1.41, *P* = 0.13		
BMI, mean(SD)	21.63 (1.89)	21.45 (1.78)	21.93 (1.69)	21.5 (1.40)	ANOVA, F = 1.63, *P* = 0.18		
Medication information							
Antipsychotics	51/55	37/47	23/29		df = 2, X^2^ = 4.69, *P* = 0.09		
Anticonvulsants	24/55	21/47	18/29		df = 2, X^2^ = 2.92, *P* = 0.23		
CPZ equivalents, mean(SD)	241.58 (93.43)	224.55 (79.90)	227.31 (64.16)		ANOVA, F = 0.519, *P* = 0.67		
Benzodiazepines	15/55	17/47	5/29		df = 2, X^2^ = 3.22, *P* = 0.20		
Lithium	22/55	19/47	12/29		df = 2, X^2^ = 4.72, *P* = 0.094		
Clinical characteristics					Man vs Eut	Dep vs Eut	Man vs Dep
Duration of illness, months, mean (SD)	70.91(59.24)	67.70 (61.91)	47.45 (34.66)		*P* = 0.140	*P* = 0.361	*P* = 1
YMRS, mean(SD)	33.78 (6.08)	8.19 (7.31)	2.78 (2.24)		*P* < 0.001	*P* < 0.001	*P* < 0.001
HDRS, mean(SD)	6.90 (8.24)	27.89 (6.61)	2.63 (3.18)		*P* < 0.001	*P* < 0.001	*P* < 0.001
Number of Previous Episodes, mean (SD)	3.55 (1.74)	3.60 (1.70)	2.90 (2.28)		*P* = 0.713	*P* = 0.654	*P* = 1
MtDNAcn, Median (range)	1.59 (1.31,2.07)	1.83 (1.22,2.31)	2.05 (1.5,2.96)	2.38 (1.77,3.24)			

*Abbreviations*: *BMI* body mass index, *SD* standard deviation,* YMRS* young mania rating scale. *HDRS* hamilton depression rating scale, *ANOVA* one-way analysis of variance, *Man* manic, *Dep* depression, *Eut* euthymic

### MtDNA copy number in subjects with BD patients and controls

**Fig. 1:**
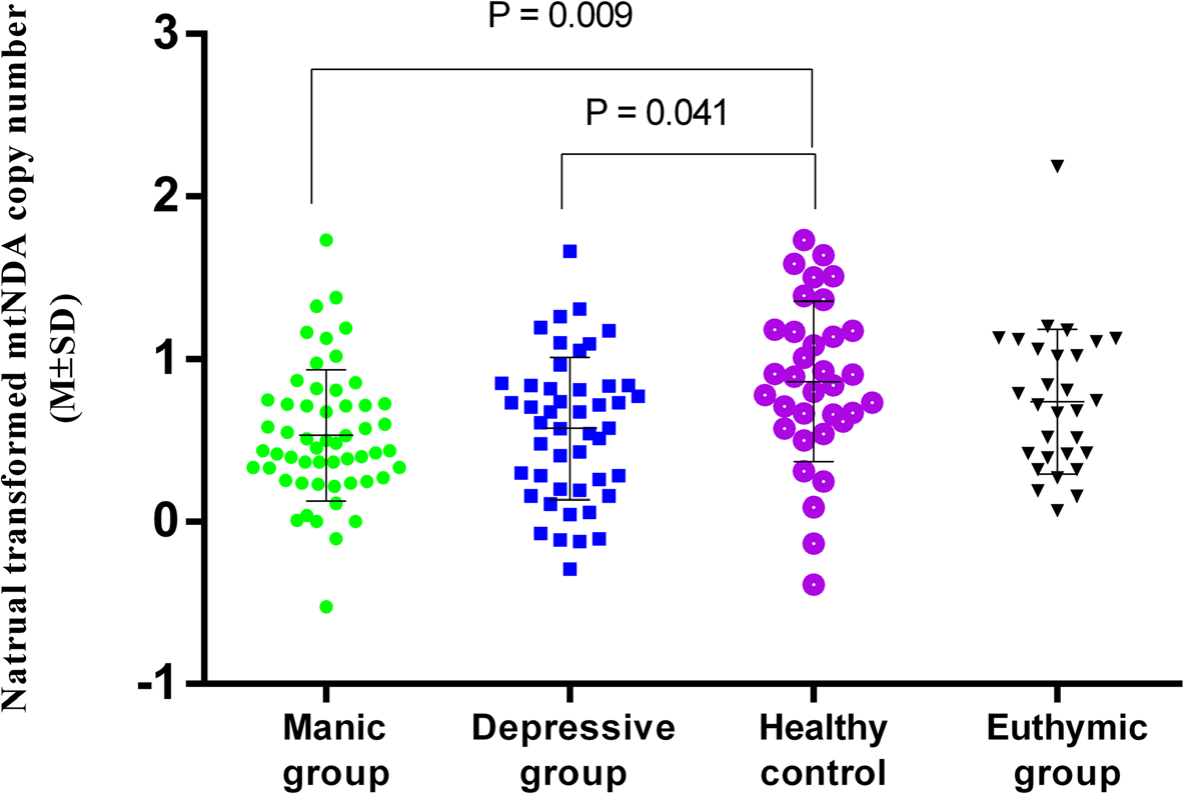
MtDNA copy number in manic, depressive, euthymic BD patients and healthy controls

After transformation, the mean mtDNAcn of four groups were 0.5 ± 0.40 (manic group), 0.57 ± 0.44 (depressed group), 0.86 ± 0.49 (euthymic group), 0.73 ± 0.45 (healthy control). The results of ANOVA showed a significant difference (*F* = 4.78, df = 3161, *P* = 0.003,*η*^*2*^ = 0.085). After controlling for age, gender, BMI, years of schooling as covariates, there remained a significant difference (*F* = 2.54, df = 6158, *P* = 0.023,*η*^*2*^ = 0.087). Besides, we found on the model, these covariates did not associate with mtDNAcn (all *P* > 0.05). The Bonferroni post hoc test revealed that, comparing with healthy group, depressed and manic groups showed a significantly lower level of mtDNAcn respectively, (*P* = 0.041,η^2^ = 0.068, s = 0.083; *P* = 0.009,*η*^*2*^ = 0.049, *s* = 0.081) (Fig. 1). No differences were detected in the mtDNAcn between manic group and depressed group (*P* > 0.05). The euthymic patients had a similar level of mtDNAcn with healthy volunteers (*P* > 0.05) (Table 2). Although the mtDNAcn of BD euthymic group were increased in comparison with manic and depressed BD group, the differences did not reach a significant level (euthymic vs. manic *P* = 0.056, euthymic vs. depressive *P* = 0.11).

**Table 2:** Effect sizes for healthy control versus patients groups and for multiple regression analyses in each patients group

	Manic group		Depressive group		Euthymic group	
	ES(SE)	*P*	ES	*P*	ES	*P*
healthy control	0.068 ^c^(0.081)	0.009^a^*	0.049^c^(0.083)	0.041^a^*	0.008^c^(0.091)	1^a^
Clinical characteristics						
Duration of illness	0.002^d^(0.001)	0.825 ^b^	0.003^d^(0.001)	0.743^b^	0.009^d^(0.004)	0.77^b^
YMRS/HDRS	0.040^d^(0.012)	0.262^b^	0.100^d^(0.016)	0.796^b^	0.259^d^(0.076)	0.212^b^
Number of Previous Episodes	0.116^d^(0.039)	0.044^b^*	0.102^d^(0.044)	0.964^b^	0.006^d^(0.065)	0.679^b^
CGI-BD-S	0.009^d^(0.067)	0.632^b^	0.02^d^(0.088)	0.724^b^	0.072^d^(0.081)	0.536^b^

*ES* effect size, *SE* standard error, *YMRS* young mania rating scale, *HDRS* hamilton depression rating scale, *CGI-BD-S* clinical global impression-bipolar disorder-severity of illness scale, ^a^adjusted *p*-values of one way ANOVA test; ^b^adjusted p-values of multiple regression analyses; ^c^Cohen’s eta squared η^2^ in ANOVA test; ^d^R^2^ in multiple regression analyses; *P < 0.05

### Association of mtDNAcn with clinical characteristics within bipolar subjects

**Fig. 2:**
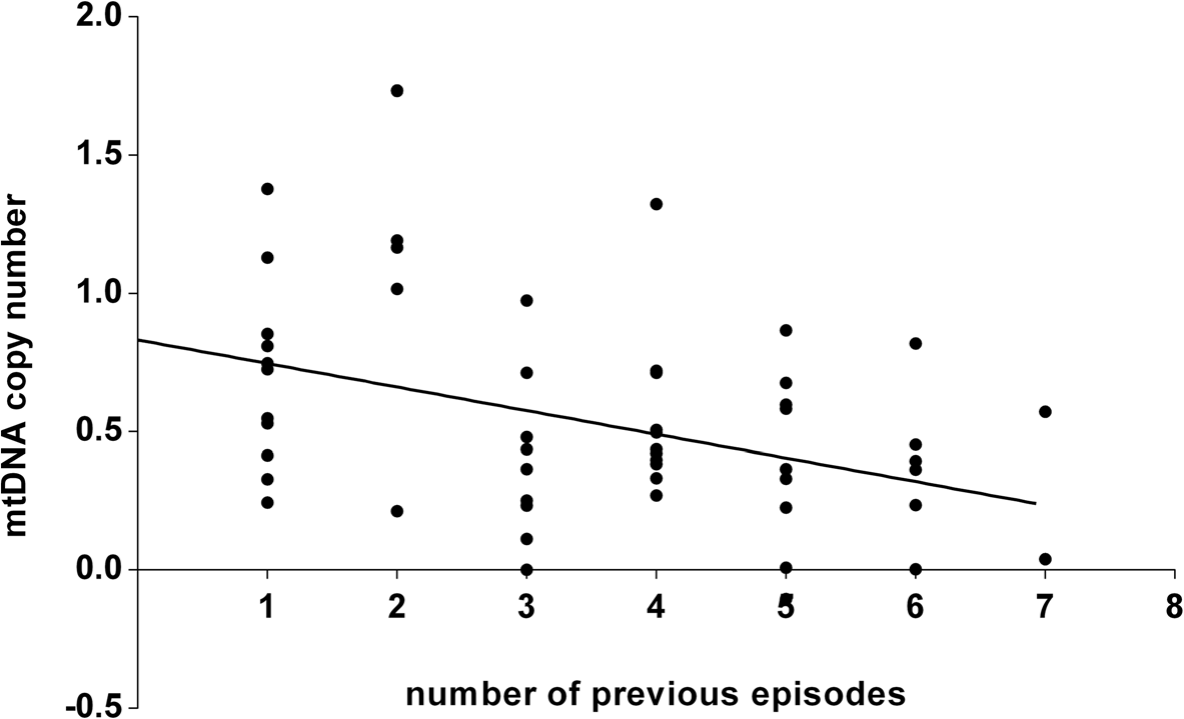
Correlation between number of previous episodes and mtDNA copy number (β = −0.341, *t* = −2.61, *p* = 0.044) within manic BD patients

Multiple regression analysis was conducted in each group to confirm the relationship between mtDNAcn and clinical information (Table 2). For the manic group, the mtDNAcn was negatively correlated with the number of relapses (*β* = − 0.341, *t* = − 2.61, *P* = 0.044, *s* = 0.039) (Fig. 2), while no similar findings happened in depressed group (*β* = 0.319, *t* = 1.19, *P* = 0.964, *s* = 0.044). The other clinical characteristics or variables had no significant association with mtDNAcn in BD patients (all *P* > 0.05).

## Discussion

To our knowledge, this is the first study to examine mtDNA content across the depressed, manic and euthymic states in BD patients. This current research has three main findings. (1) The acute BD patients had markedly lower mtDNA amount than healthy controls, after controlling covariates; there were no differences in mtDNAcn between manic patients and depressed patients. (2) The euthymic group showed a comparable content of mtDNA with healthy control group. (3) The mtDNAcn was inversely correlated with the number of relapses in BD subjects during manic episodes.

Change of mtDNAcn is an important sensitive index of mitochondrial dysfunction and oxidative stress [29]. Our findings indicated that there may be explicit mitochondrial impairments during episodes of BD. In the current study, we firstly reported decreased mtDNA amount in peripheral leukocytes of BD patients undergoing manic episodes. Our data suggested, manic group may display a similar level of mtDNA with depressive group. In contrast, there was nearly normal level of mtDNA in euthymic patients. Hence, we speculate that, leukocyte mtDNA content may change with mood states in BD. Nicod et al. suggested the mtDNA content of patients changed with time during 8-week treatment in major depression [30]. Acute patients could recover after treatment, and gradually transit to euthymic states. According to the findings and clinical observations above, the mtDNAcn of acute patients in peripheral blood might be gradually restored to some extent as time goes on.

The results of prior data on mtDNAcn in peripheral leukocytes and brain tissues of mood disorder were not consistent. Compatible with our result, de Sousa et al. showed a slight decreased mtDNAcn in depressive patients with BD type I, although the differences just failed to be significant (*P* = 0.05), due to a small sample (*N* = 7) [21]. Chang et al. described that mtDNAcn was still significantly lower in the BD euthymic patients [20]. One plausible explanation is that mtDNAcn was negatively correlated with age in BD [21], and their subjects were older than ours. Accumulated evidences demonstrate BD may be an accelerated aging disease [31]. Aging was also reported to be associated with the downregulation of mtDNA-associated genes and a decline level of mtDNAcn [32]. Therefor accelerated aging in BD may affect the levels of mtDNA. According to threshold hypothesis of mtDNAcn control [12], low mtDNAcn triggers the upregulation of mtDNA replication and high mtDNAcn triggers the machinery to degradate mtDNA. Taken together, low threshold and accelerated aging may induce the decrease of mtDNAcn. Regarding mtDNAcn in postmortem brains, three out of four studies reported a negative result in BD [16–19]. It was not clear about what kinds of mood states that those subjects suffered from. Thus, the comparison cannot be made with ours. Additional, another innegligible factor is using different tissues or cells in studies, which may confound the results. .

MtDNA polymerase gamma (POLG) is the only one polymerase responsible for the mtDNA replication. POLG is prone to oxidative damage,and its deleterious variants were suggested as a risk for BD [33]. Munkholm et al. revealed downregulation of POLG expression in BD across mania, hypomania, depression or mixed states in peripheral leukocyte [34]. We suppose that POLG downregulation in acute phases of BD may be another important point of resulting in decrease of mtDNAcn.

There are also several evidences for abnormal mtDNA amounts in major depression [35–38]. A large sample multicenter study reported patients with major depression showed increased mtDNA level than controls in saliva and blood [37]. Two studies found a lower peripheral mtDNA level in both depressive and euthymic states [35, 38]. Nicod et al. suggested mtDNA content of patients changed with time in major depression [30].Overall it is not consistent about changes of mtDNA content in major depression and bipolar depression. However all studies indicate the importance of mtDNA in mood disorder. Future research is warranted to explore the relationship in different states of mood disorders.

The relationship observed between mtDNAcn and the number of episodes in manic patients deserves attention. In the current study, we found that mtDNA content was negatively correlated with the number of episodes in BD patients. BD patients often experience recurrent episodes or relapses which appear to cause chronicity of this illness and vulnerability for high stress response [39]. The subjects suffering manic episode are at a high stress level [40]. The “sensitivity” state of oxidative stress may accelerate illness course, and cause cell deficits as well as rapid decline of mitochondrial function.. However, similar findings were not found in the depressive subjects of this study. Hence, the results should be interpreted cautiously, due to the heterogeneity and complexity of BD.

Although antipsychotic agents and mood stabilizers were reported to exert some effect on mitochondrial function [4, 41–43]. de Sousa and our previous studies showed that six or eight weeks lithium or risperidone treatment have no significant effect on mtDNAcn in subjects with psychiatric disorders [13, 21]. Accordingly, we suppose the mtDNAcn of the acute patients would make no significant changes after short–term treatment. Whereas our euthymic patients were medically stabilized for more than 6 months, and may have enough time to recover from various damage in cell.

There are several limitations to be considered. Since we didn’t have more clinical data, including smoking status, glucose intolerance, and serum lipid, the possible influence of these variables cannot be completely excluded. Although using peripheral blood is more convenient and less invasive to quantify mtDNAcn, these finding cannot be extrapolated mechanically to other tissues. In addition, only mtDNAcn was measured in this cross-sectional data, it does not indicate any information about causal relationship between oxidative stress, mtDNA variations, and BD. More biological parameters and longitudinal studies regarding mitochondria involved in the pathophysiology of BD are warranted in future.

## Conclusion

This study provides novel observations that Leukocyte mtDNA content was significantly decreased in BD patients suffering manic and depressive episodes, as compared with healthy controls. Our results also showed mtDNAcn was negatively correlated with the number of relapses in manic BD patients. Mitochondrial abnormalities in acute patients might involve in the pathophysiology of BD. The findings maybe promote a further understanding on the role of mitochondria in BD. Although our data indicated the mtDNAcn might change with mood states, the underlying mechanisms are complex and still unclear. More effort for a better explanation of the processes engaged in BD is required in further investigation.

## Additional file

Additional file 1: Excluded neurological diseases and other serious physical conditions in the study. (DOCX 15 kb)

## Abbreviations

ANCOVA: One-way analysis of covariance
ANOVA: one-way analysis of variance
ATP: adenosine triphosphate
BD: Bipolar disorder
BMI: Body Mass Index
CGI-BD-S: Clinical Global Impression-Bipolar Disorder-Severity of Illness Scale
CPZ: chlorpromazine
Dep: Depression
DSM-IV: Diagnostic and Statistical Manual of Mental Disorders Fourth Edition
Eut: Euthymic
HAM-D: Hamilton Depression Scale
Man: Manic
mtDNAcn: mitochondrial DNA
mtDNAcn: Mitochondrial DNA copy number
POLG: polymerase gamma
ROS: reactive oxygen species
SCID-IV: Structured Clinical Interview for DSM- IV Axis I Disorders
SD: Standard Deviation
YMRS: Young Mania Rating Scale
