# Supplementary material for: Differential mitochondrial DNA copy number in three mood states of bipolar disorder
Source: BMC Psychiatry. 2018 May 25;18:149. doi: 10.1186/s12888-018-1717-8 (PMC5970444; doi:10.1186/s12888-018-1717-8)
Supplement: Supplementary file 1 — Excluded neurological diseases and other serious physical conditions in the study. (DOCX 15 kb) [file 12888_2018_1717_MOESM1_ESM.docx]

Additional file 1

Excluded neurological diseases and other serious physical conditions in the study

List of excluded neurological diseases and other serious medical conditions

(1). Excluded neurological diseases:

Brain damage;

Seizure disorders;

Movement disorders of the central and peripheral nervous system such as Parkinson's disease, Essential tremor, Amyotrophic lateral sclerosis, Tourette's syndrome;

Degenerative disorders of the nervous system such as Parkinson's disease, essential tremor, Huntington's disease, Alzheimer's disease, multiple sclerosis and organic psychosis;

Stroke (cerebrovascular accidents, cerebrovascular attack);

Tumors of the nervous system (e.g. cancer);

Infections of the brain;

Other known neurological diseases obtained from individuals or their family.

(2). Excluded serious physical condition:

Hypertension, diabetes, hematological system diseases, heart diseases, other known endocrine and cardiovascular diseases, infectious diseases, other known serious physical diseases.
